# A comparative study of ribosomal proteins: linkage between amino acid distribution and ribosomal assembly

**DOI:** 10.1186/2046-1682-6-13

**Published:** 2013-10-23

**Authors:** Brittany Burton Lott, Yongmei Wang, Takuya Nakazato

**Affiliations:** 1 Department of Chemistry, The University of Memphis, 38152 Memphis TN, USA; 2 Department of Bioinformatics, The University of Memphis, 38152 Memphis TN, USA; 3 Department of Biological Sciences, The University of Memphis, 38152 Memphis TN, USA

**Keywords:** Ribosomal assembly, Amino acid compositions, Electrostatic interactions, Adaptation, Protein/RNA contacts, Thermostability, r-proteins, 30S subunit

## Abstract

**Background:**

Assembly of the ribosome from its protein and RNA constituents must occur quickly and efficiently in order to synthesize the proteins necessary for all cellular activity. Since the early 1960’s, certain characteristics of possible assembly pathways have been elucidated, yet the mechanisms that govern the precise recognition events remain unclear.

We utilize a comparative analysis to investigate the amino acid composition of ribosomal proteins (r-proteins) with respect to their role in the assembly process. We compared small subunit (30S) r-protein sequences to those of other housekeeping proteins from 560 bacterial species and searched for correlations between r-protein amino acid content and factors such as assembly binding order, environmental growth temperature, protein size, and contact with ribosomal RNA (rRNA) in the 30S complex.

**Results:**

We find r-proteins have a significantly high percent of positive residues, which are highly represented at rRNA contact sites. An inverse correlation between the percent of positive residues and r-protein size was identified and is mainly due to the content of Lysine residues, rather than Arginine. Nearly all r-proteins carry a net positive charge, but no statistical correlation between the net charge and the binding order was detected. Thermophilic (high-temperature) r-proteins contain increased Arginine, Isoleucine, and Tyrosine, and decreased Serine and Threonine compared to mesophilic (lower-temperature), reflecting a known distinction between thermophiles and mesophiles, possibly to account for protein thermostability. However, this difference in amino acid content does not extend to rRNA contact sites, as the proportions of thermophilic and mesophilic contact residues are not significantly different.

**Conclusions:**

Given the significantly higher level of positively charged residues in r-proteins and at contact sites, we conclude that ribosome assembly relies heavily on an electrostatic component of interaction. However, the binding order of r-proteins in assembly does not appear to depend on these electrostatics interactions. Additionally, because thermophiles and mesophiles exhibit significantly different amino acid compositions in their sequences but not in the identities of contact sites, we conclude that this electrostatic component of interaction is insensitive to temperature and is not the determining factor differentiating the temperature sensitivity of ribosome assembly.

## Background

Ribosomes are the transient macromolecular machines that synthesize proteins in all living organisms. They are composed of ribosomal RNA (rRNA) and ribosomal proteins (r-proteins), which self-assemble into functional units. The bacterial ribosome is made of two asymmetrical subunits: the larger 50S and the smaller 30S. This study focuses on the assembly of the 30S subunit. The efficient and accurate self-assembly of the ribosome *in vivo* is essential because new ribosomes and proteins must be produced in order for cells to grow. It is estimated that approximately 60% of all cellular transcriptional activities have been attributed to the synthesis of rRNA in a rapidly growing cell [1] and 40% of the total energy of an *Escherichia Coli* cell is directed toward the synthesis of proteins [2]. Assembly has been studied extensively, both computationally and experimentally, and is known to require the orchestration of both rRNA folding and r-protein binding. Previous investigations provide evidence of an ordered, cooperative protein binding/RNA folding assembly mechanism [3-5], conserved structures and sequences [6-11], and the employment of electrostatics interactions [12-14]. A detailed assembly map describing the sequential and interdependent binding of r-proteins [4] classified r-proteins as primary, secondary, and tertiary binders, depending on their ability to bind to 16S rRNA: primary proteins bind to bare rRNA, secondary proteins can bind to 16S rRNA after at least one primary protein has already bound, and tertiary proteins require at least one primary and one secondary protein [15]. Additionally, r-proteins were named S1, S2, S3, etc., in the general order of decreasing size; that is, S1 is the largest ribosomal protein and S21 the smallest [16,17].

Because r-proteins strongly interact with negatively charged rRNA to form a functional complex, one might expect that r-proteins exhibit characteristic amino acid composition and distribution within the protein structures that reflect their electrostatic interactions. For instance, it is known that r-proteins generally carry net positive charges [13,14], and we previously analyzed the crystal structures of two bacterial ribosomes and found that most *E. coli* and *Thermus thermophilus* r-proteins not only carry net positive charges, but their percentages of positively charged residues are actually above the average expected for a typical protein [12]. We also demonstrated that these positively charged residues tend to be concentrated in areas of the protein that are in contact with rRNA. These observations are consistent with the hypothesis that positively charged residues facilitate and stabilize r-protein binding to the negatively charged rRNA. Because these studies encompassed such a small portion of the bacterial kingdom, the investigation of r-proteins from a large number of species is needed to more definitively describe the nature of this trend. To date, however, large-scale analyses comparing the ribosomal components from many species have focused on the use of rRNA, r-proteins, or ribosomal DNA to determine species relatedness or construct phylogenetic trees [18-21] rather than attempting to shed light on the universal mechanisms of ribosome assembly.

Temperature has profound effects on the rates of biological reactions and the structures of molecules, including proteins. Because the structure and function of a protein are ultimately controlled by its makeup of amino acids, one would expect proteins from thermophilic species to have different amino acid composition from those of mesophilic species. In accordance, several large-scale thermostability studies have detected differences in protein residues, such as thermophiles exhibiting an increased occurrence of charged residues, decreased incidence of polar and uncharged residues, a reduction in hydrophobic surface of the protein, larger numbers of hydrogen bonds, ion pairs, and disulfide bridges or hydrophobic and aromatic interactions, an increased protein compactness, and changes in surface charge distribution and helix dipole stabilization [22-30]. While the majority of these previous protein thermostability analyses have focused primarily on non-ribosomal protein samples, one [30] mentioned that the trends were not significantly changed when r-proteins were excluded from analysis. Some studies have focused on ribosomal components in light of thermal adaptation, identifying a positive correlation between the guanine and cytosine content in rRNA genes and the species growth temperature [31], and demonstrating that the binding affinity of r-protein S8 with its rRNA binding site increases with growth temperature among related bacterial species [32]. Additionally, it has been shown [33,34] that subunits from a thermophilic Archaea can form functionally active hybrids with eukaryotic yeast subunits (i.e. the small subunit from one species and the large from another), whereas no such particles formed between the subunits from a mesophile and yeast, suggesting that there is at least some structural similarity between ribosomes from thermophilic bacteria and eukaryotic species. One study [35] compared the stability of the entire ribosome structure in mesophiles and thermophiles, showing that thermophilic ribosomes are generally nonfunctional at low temperatures and hypothesizing that thermophilic ribosomes might be prohibitively rigid at low temperatures in order to be functionally flexible at their optimal growth temperatures. This is in agreement with a report from “melting” and unfolding studies, indicating thermophilic ribosomes are more “durable” than those isolated from mesophiles [36]. Similarly, it has been shown that the individual components of a thermophilic ribosome are less stable than the completely assembled ribosome [37]. In our previous study [12], we observed that r-proteins of the thermophilic *T. thermophilus* generally have higher net positive charges than those of mesophilic *E. coli*, possibly implicating differing roles of certain amino acids in the structure or function of thermophilic and mesophilic r-proteins. While these thermostability studies have enriched the current understanding of ribosome structures and temperature-sensitive characteristics in a variety of species, details regarding the contributions of individual amino acids to the ribosome’s accurate self-assembly mechanisms and the factors that differentiate species’ ability to create thermostable complexes within certain temperature ranges remain uncertain.

In the current study, we extend our previous work to include 560 different bacterial species (listed in Additional file 1) to test whether the reported trends hold for prokaryotes in general. For this purpose, we employ a comparative approach where association is tested between the average occurrence of each amino acid and the members of two categories of house-keeping bacterial proteins: ribosomal proteins and non-ribosomal proteins. Additionally, we compare r-protein sequences from mesophilic and thermophilic species to examine how amino acid composition and distribution might affect ribosome assembly at differing environmental temperatures.

## Results and discussion

### R-proteins contain higher levels of positively charged residues than other soluble protein families

**Figure 1:**
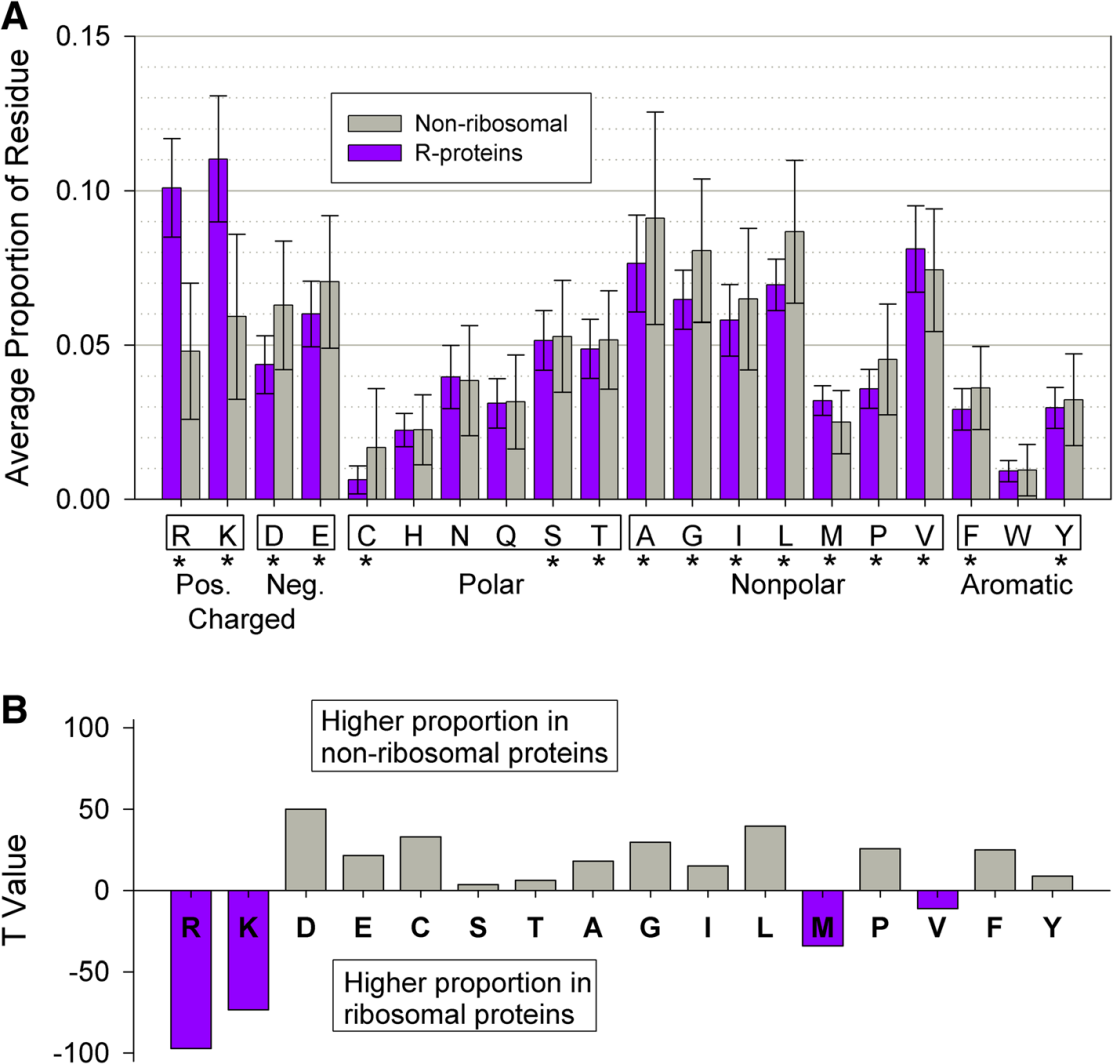
**Student’s T-test shows significant differences between ribosomal and non-ribosomal proteins. (A)** Average amino acids compositions found in ribosomal proteins (purple) and non-ribosomal proteins (light blue) samples. Asterisks indicate a statistically significant difference between the two averages; error bars are ±σ. **(B)**: T-values from Student’s t-tests for the amino acids compositions with significant difference between the two groups. The magnitude of the bar represents the relative difference between the two means and the direction of the bar (up or down) indicates which protein sample contains the larger proportion of that residue. A positive T-value indicates a higher proportion of that residue found in the non-ribosomal sample, whereas a negative T-value corresponds to a higher proportion of that residue found in ribosomal proteins.

To test whether the unusually high proportion of positive amino acids (Arginine (Arg, R) and Lysine (Lys, K)), identified in our recent study of *E. coli* and *T. thermophilus* is a general pattern among bacteria, we compared the proportion of each amino acid between ribosomal and other house-keeping, non-ribosomal proteins from 560 species (Figure 1A). For each species, we calculated the percentage of each amino acid across all 30S ribosomal protein sequences and in each of the 15 non-ribosomal protein families. Student’s paired sample t-tests revealed significant differences between ribosomal proteins and non-ribosomal families in the proportions of all amino acids except for Histidine, Asparagine, Glutamine, and Tryptophan (H, N, Q, and W; Figure 1A; see Additional file 2 for statistical values). In ribosomal proteins, the positive residues Arg and Lys make up the largest proportions of the sequences, at 10% and 11%, respectively, whereas the non-ribosomal proteins have 4.7% Arg and 5.9% Lys. Many other amino acids generally exhibited significantly higher proportions among non-ribosomal proteins, but it is likely that these differences are largely a consequence of the much lower proportions of Arginine and Lysine. Therefore, it appears that an unusually high proportion of positive amino acids is a defining characteristic of prokaryotic r-proteins. It is worth noting that, in non-ribosomal proteins, the average proportions of the acidic, negatively charged residues at physiological pH (Aspartic Acid (Asp, D) and Glutamic Acid (Glu, E)) are roughly equivalent to the average proportions of basic, positively charged residues (Arg, Lys). This results in, on average, a neutral net charge for those proteins. However, for r-proteins, the percentages of positively charged basic residues are considerably larger than for negatively charged acidic residues. This is in agreement with the previously reported net positive charges for r-proteins and indicative of the role of electrostatic attractions between r-proteins and negatively charged rRNA during ribosome assembly.

Figure 1B shows the magnitude and direction of the significant differences in the amino acid distributions for the two samples of proteins, represented by their t-test values. The height of the bar represents the relative difference in the sample means and its direction indicates which protein sample contains the larger proportion of that residue. Positive T-test values indicate a higher proportion of that residue was found in the non-ribosomal sample, whereas negative values correspond to a higher percentage in r-proteins. It is well documented that ribosomal proteins contain high levels of these positively charged residues, and the marked difference shown here clearly implicates an important electrostatics feature of r-proteins in contrast to proteins whose functions do not rely heavily on charge-charge interactions [12-14]. This result solidifies our earlier observation that ribosomal proteins have higher proportions of positively charged residues and that the assembly between ribosomal proteins and rRNA includes an important electrostatic component, a notion that has also been suggested by other studies [38]. It is evident that these amino acids play an important role in the assembly process, attracting positively charged r-proteins to negatively charged rRNA across possibly long distances to initiate the assembly process. While this line of reasoning is not novel, the overwhelming significance of positively charged residue content indicates our amino acid composition database imparts a rational view of r-protein make-up, and provides the foundation for the rest of the current study. This observation prompted further investigation into the large database of r-protein sequences, particularly with regard to the roles of these amino acids in the electrostatics component of ribosome assembly.

Because increased temperature is known to denature and destabilize biological molecules, yet thermophilic bacteria synthesize and assemble ribosome components that maintain functionality at consistently high environmental temperatures [36,37], we analyzed r-protein amino acid composition to test whether the amino acid make-up plays a role in the thermostability of the r-proteins. To this end, we utilized a comparative approach where association was tested between the growth temperature preferences of a large number of thermophilic and mesophilic bacterial species and the proportion of each amino acid in the r-protein sequences, specifically focusing on amino acid compositional differences associated with thermophilicity. We obtained three types of information for the 560 species in our database: growth temperature preference data, 30S ribosomal protein sequences from at least one r-protein, and 16S ribosomal DNA sequences (to determine species relatedness). The vast majority consisted of mesophiles and only 40 were identified as thermophiles. Phylogenetic analysis of these species indicated that thermophiles are not evenly distributed in the bacterial phylogenetic tree: they tended to cluster in several branches, especially in the orders Aquificales, Thermoanaerobacterales, and Thermotogales (Additional file 1).

The phylogenetic clustering of thermophiles in our sample necessitated us to employ a method to control for the phylogenetic dependence and avoid bias when assessing the association between growth temperature preference and ribosomal amino acid composition. Because closely related samples are expected to show similar traits such as amino acid composition and growth temperature preference, a significant association can simply be a result of phylogenetic relatedness rather than adaptation to similar environmental conditions. To circumvent this problem, we applied Phylogenetic Independent Contrast (PIC [39,40]), which assesses the statistical significance of correlations between variables while controlling for the phylogenetic relatedness among samples. In this way, a significant correlation implies that the differences in amino acid composition between thermophiles and mesophiles are due to adaptation to different temperature environments and not due to mere species relatedness. It should be noted, however, that PIC is conservative, because it fails to detect significant adaptive changes that accompany significant phylogenetic dependence.

**Figure 2:**
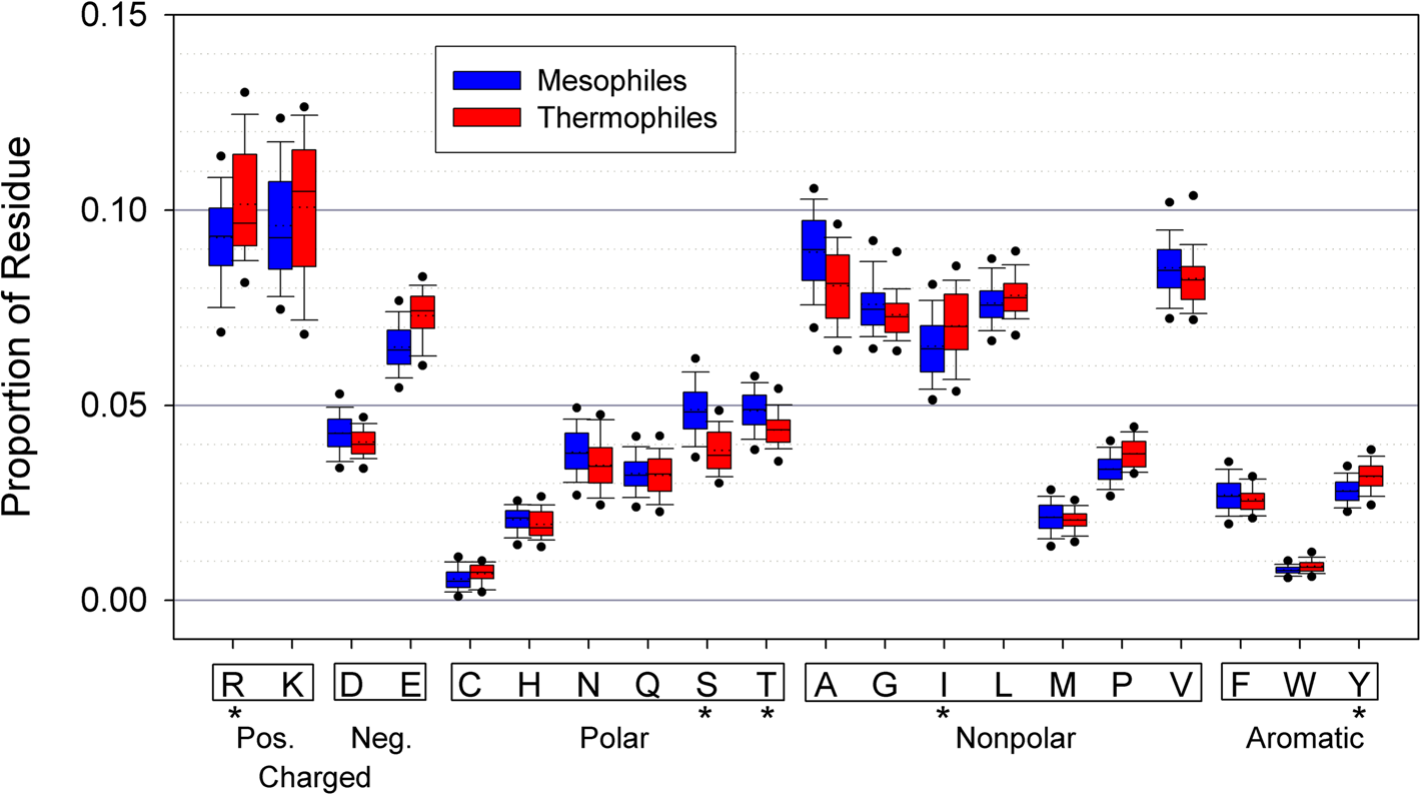
**R-protein amino acid compositions exhibit typical thermostability differences.** Thermophilic r-proteins (red) contain higher percentages of Arginine (R), Isoleucine (I), and Tyrosine (Y), and lower percentages of Serine (S) and Threonine (T) than mesophilic r-proteins (blue). These differences are generally consistent with typical differences among thermophilic and mesophilic proteins and are estimated to function in the thermostability of the protein. In the box-and-whisker representation, the lower and upper circles represent the 5th and 95th percentiles, respectively, and the lower and upper whiskers the 10th and 90th. The colored regions mark the middle 50% of the samples (25th to 75th percentile), with a solid line representing the median and a dotted line the mean. Asterisks mark the amino acids that show a statistically significant difference between mesophilic and thermophilic species.

PIC analyses revealed that, at the level of the entire 30S subunit, thermophiles are comprised of significantly lower proportions of polar Serine (S) and Threonine (T) residues and higher proportions of positively charged Arginine (R), nonpolar Isoleucine (I), and aromatic Tyrosine (Y) (Figure 2; according to at least one statistical significance test at α = 0.01; see Methods and Additional file 3). Other differences in mean values between the two groups, though they may appear somewhat large, are not significant according to sign test or t-tests. These results are largely consistent with other thermostability studies (reviewed in Ref. [29]), which have identified an increase in R and Y levels and a decrease in C and T levels in thermophiles. It is worth mentioning that a seeming discrepancy in our report merely involves similar but different polar residues: we report a significant difference in Serine (CH_2_OH side chain) instead of Cysteine (CH_2_SH side chain), as found in other studies. The general trends we observed via PIC also match previous thermostability reports: thermophiles contain significantly higher proportions of positive residues and lower proportions of polar residues than mesophiles (at α = 0.01). These tendencies likely reflect the need for stronger interactions at higher temperatures [23,25,29]. On the other hand, no significant directional biases were detected for negative, nonpolar, and aromatic residues.

### Positively charged residues correlate with protein size but not binding order

**Figure 3:**
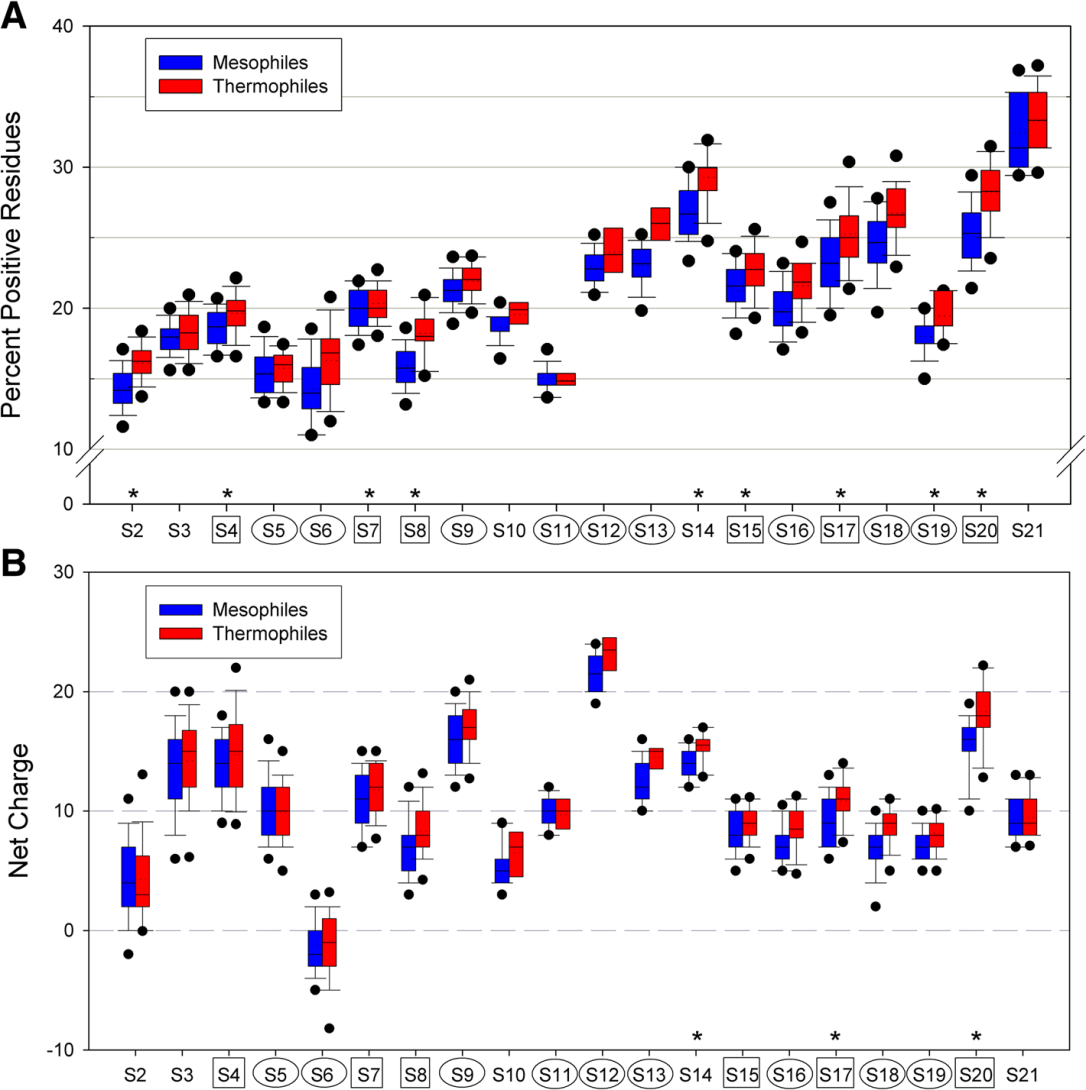
**Percentage of positively charged residues correlates with protein size but net charges do not.** All thermophilic r-proteins except S11 contain a higher percentage of positively charged residues than their mesophilic homologs **(A)**, and, for some proteins, including all six primary proteins, this difference is statistically significant. R-proteins generally have a net positive charge **(B)**, and thermophiles typically have a higher average charge than mesophiles. For three proteins, this difference is significant. The box-and-whisker plots are represented as in Figure 2.

Because primary binding proteins bind to the bare, negatively charged RNA during ribosomal assembly and the binding electrostatics of subsequent proteins might differ due to the presence of already-bound r-proteins, one might expect that primary r-proteins have higher proportions of positive residues than secondary and tertiary proteins (which are unlikely to bind before primary proteins) or that primary proteins may have higher net positive charges. Correlations with respect to protein assembly order between the proportion of positive residues and the net charges on the proteins were tested. However, we did not find evidence for higher proportions of positive charges in primary proteins (Figure 3A; see Panel A in Additional file 4 for a visualization of proportion of positive charges according to protein binding order), as Student’s t-test comparing the mean proportions of positive residues between primary and secondary/tertiary r-proteins was not significant (t_15_ = −0.207, two-tailed p = 0.839), suggesting that binding order is not influenced by fractions of positively charged amino acid of r-proteins. Statistical tests of association between net charge and binding order also revealed no observable correlation (Spearman’s Rank correlation ρ = 0.190; Figure 3B; see Panel B in Additional file 4 for a visualization of net charges according to protein binding order). Because no relationship between the order in which r-proteins attach to the rRNA during assembly (primary versus secondary versus tertiary) and the content of positive residues or the total protein charge was detected, it is likely that binding order is governed by mechanisms other than simple electrostatics interactions with the RNA, possibly the availability of the binding sites on RNA.

To determine whether increased temperature affects the relative proportions of amino acids in bacterial r-proteins regarding binding order, we analyzed the amino acid compositions according to species optimal growth temperature (see Methods). For positive residues (Figure 3A), all r-proteins except S11 showed higher mean percent residues in thermophiles than mesophiles, whereas for polar residues (Panel A in Additional file 5), all thermophilic proteins showed lower mean percent residues than their mesophilic counterparts. This suggests that the preference of positive residues at the expense of polar residues among thermophiles applies nearly universally to all r-proteins of the 30S subunit, as has also been evidenced in other protein families [23]. However, only some r-proteins, including all primary binding proteins, tended to show statistically significant differences between the two temperature-based groups for positive residues (Figure 3A). Few proteins showed statistical differences for other categories, according to no discernible pattern (Additional file 5; see Additional file 6 for summaries of statistical test results). These trends suggest that thermophiles tend to prefer positive residues and avoid polar residues across all r-proteins, and this trend is somewhat pronounced for primary binding proteins. Average net charges of individual r-proteins of thermophilic species are higher than mesophilic, except for S2, but only three proteins (S14, S17, and S20) show differences that are statistically significant according to PIC analysis (Figure 3B; see Additional file 7 for statistics).

**Figure 4:**
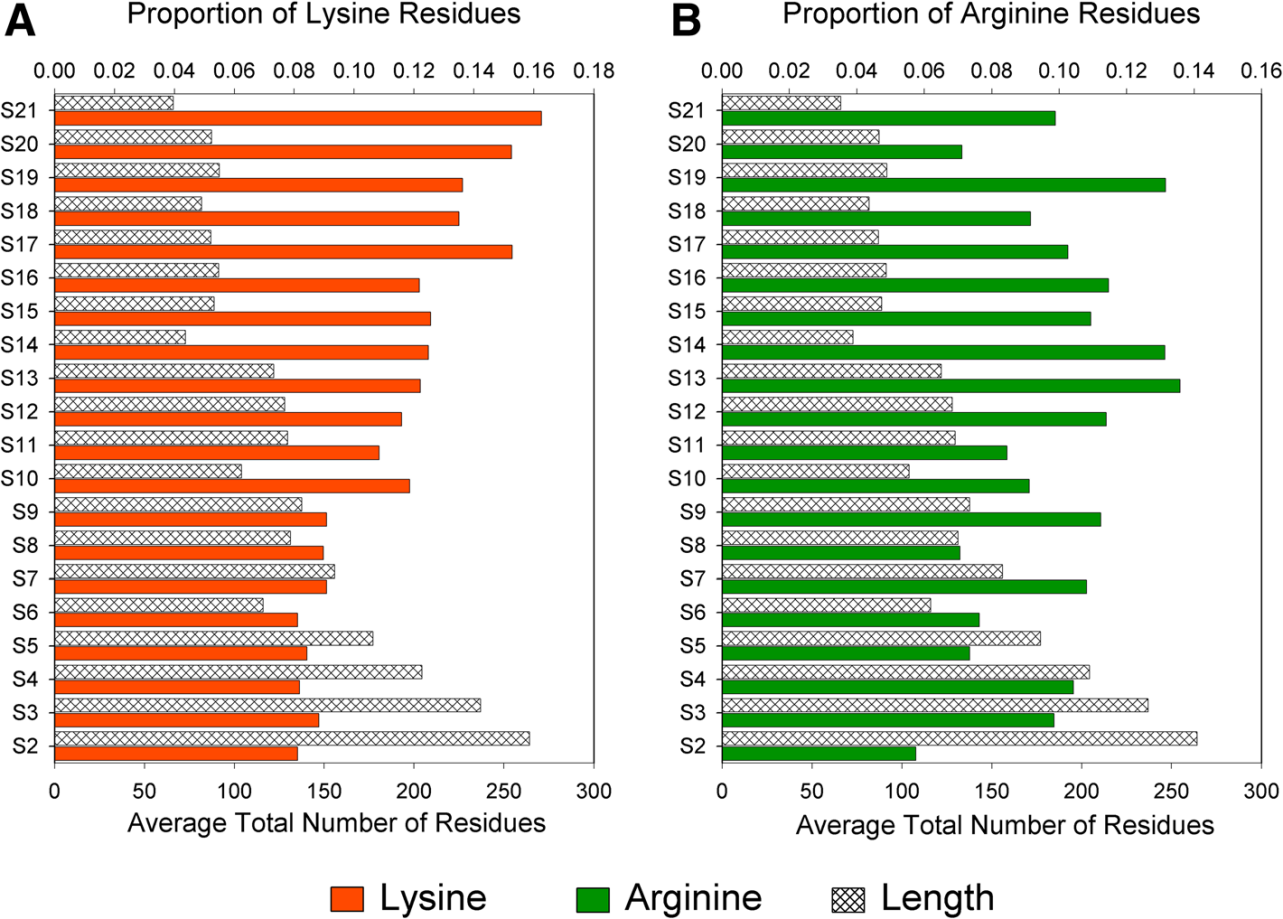
**Proportion of Lysine residues correlates with average protein length but Arginine does not.** Lysine **(A)** shows a highly significant negative correlation with protein length (Spearman’s rank correlation: ρ = −0.802, p=2.60x10^-5^), whereas Arginine **(B)** shows a weaker correlation with no statistical significance (ρ = −0.484, p=0.032).

Upon analyzing the data shown in Figure 3A, we noticed a second general trend: increasing percentage of positively charged residue from S2 to S21. Because r-proteins are named in order of decreasing size, this relationship appears to be between positive charges and protein length. We have already shown the high incidence of positively charged residues is an important feature of all ribosomal proteins, and here we find that smaller proteins tend to have higher proportions of them. Interestingly, this relationship appears to be due to Lysine content rather than Arginine content (Figure 4; Spearman’s rank correlation ρ = −0.802, p = 2.60×10^-5^ for Lys, ρ = −0.484, p = 0.032 for Arg; see Additional file 8 and Additional file 9). This result is intriguing, as it provides evidence that amino acids usually considered chemically equivalent are not necessarily used interchangeably in bacterial proteins. It hints at differential functions of chemically similar residues, even in their roles in the electrostatics component of ribosome assembly. From the current study, it is unclear why Arg does not participate in this trend. We also identified a positive correlation between percent of Glycine and increasing protein size (G; Spearman’s rank correlation, ρ = 0.657, p = 0.002), but none were detected for other residues. Neither was a correlation found between average net protein charge and average length (see Figure 3B; Spearman’s Rank correlation ρ = 0.239), indicating that this is truly an association involving only the content of the Lys residue.

### R-protein RNA contact sites are enriched with positively charged residues

**Figure 5:**
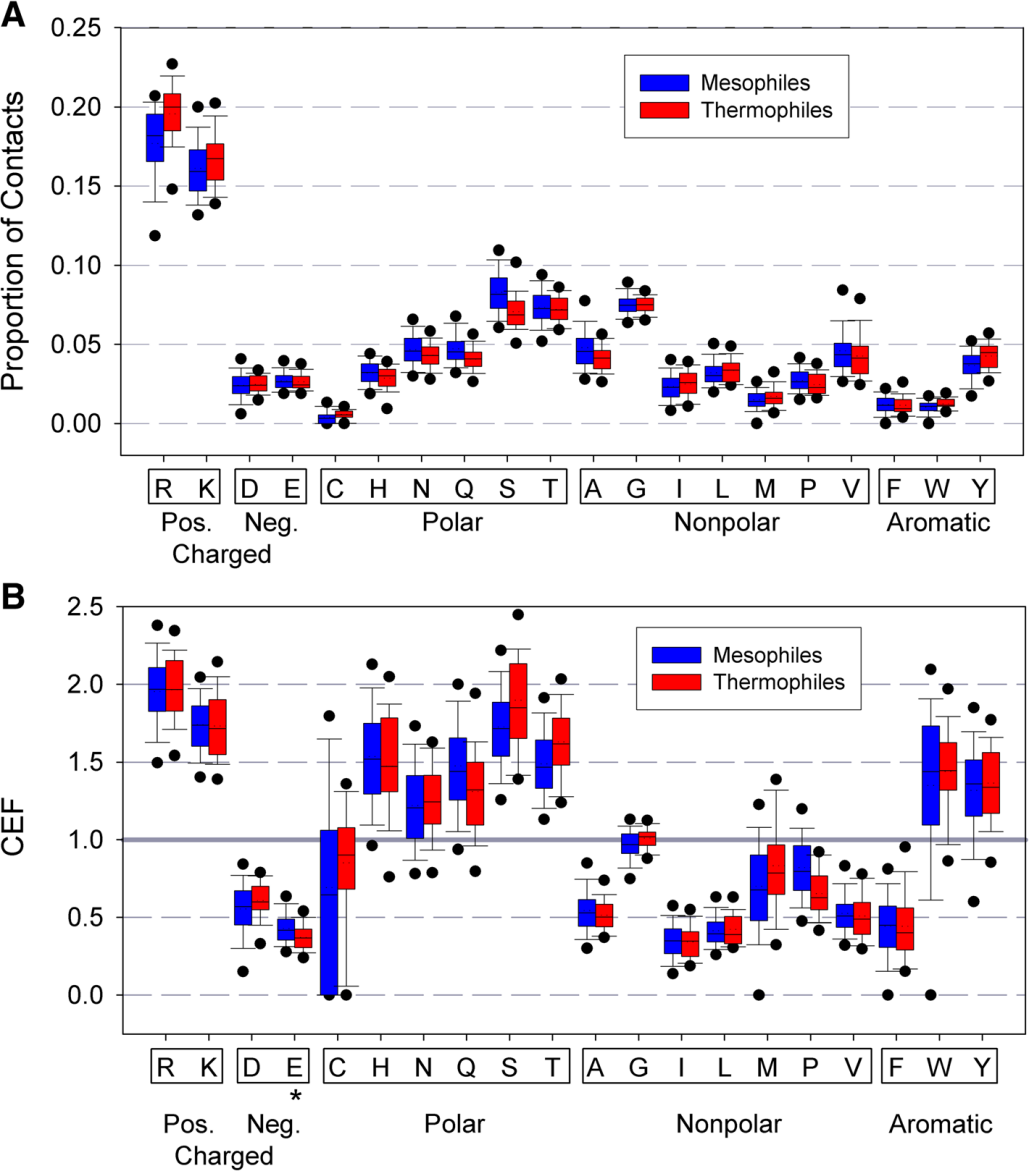
**Generally, contact residue identities are not statistically different between mesophiles and thermophiles. (A)** R-proteins (purple) show reasonable distributions of amino acids at contact sites: positively charged and polar residues are likely to interact with the negatively charged rRNA, so high CEF are expected. A CEF > 1 indicates a high prevalence for that amino acid to be located at a contact site; a CEF < 1 indicates a deficiency; a CEF ~ 1 indicates no preference for that amino acid to be located at contact versus non-contact sites. Asterisks indicate the residues whose CEF deviate significantly from 1 (Student’s t-test, α=0.01). The box-and-whisker plots are represented as in Figure 2. **(B)** CEF for the amino acids at the estimated rRNA contact sites for mesophilic (blue) and thermophilic (red) r-proteins. Only Glutamic acid, E, shows a CEF mean that is statistically different between mesophiles and thermophiles. This is an intriguing finding, considering the significant differences in overall amino acid composition, as shown in Figure 2.

We have just shown that r-proteins contain a significantly higher percentage of positively charged residues than other bacterial proteins, which is likely indicative of their importance in some fashion. If these positively charged residues are recruited for the purpose of assembly, one might expect them to be concentrated at the protein sites contacting rRNA. In our previous work, we used the X-ray crystal structures of 30S for two bacterial species, *E. coli* [PDB: 2AVY] [41] and *T. thermophilus* [PDB: 1J5E] [42], to identify contact residues as amino acids containing at least one atom within 3.5Å of any nucleic acid atom. We showed that these contact sites were indeed enriched with positively charged residues: 39% and 46% of contacts were made by positively charged residues for *E. coli* and *T. thermophilus*, respectively [12]. In order to see if such trends are true for bacterial species in general, in the current study we computed two descriptors of r-protein contact residue distributions. First, we calculated the amino acid composition at contacts between r-proteins with RNA across all r-proteins from all 560 species. This was calculated via R_c_/C, where R_c_ is the number of contacts made by a given amino acid and C is the estimated number of total contacts with RNA in the fully assembled 30S subunit (see Methods). These proportions (shown in Figure 5B) clearly show the elevated representation of positive residues at r-protein contact sites: on average, over a third of contacts are made by positively charged residues (34% for the sum of R and K in thermophiles and 36% in mesophiles). Although the mean percent of positive residues as contacts is higher in thermophile than in mesophiles, PIC analysis reveals the difference is not statistically significant. This is in contrast to the overall amino acid composition between mesophiles and thermophiles, for which the percent of R present in thermophiles is statistically different from that in mesophiles. Other differences between mean proportions of contact residue identities in mesophilic and thermophilic r-proteins are similar to or only slightly different from the overall distribution of amino acids (as seen Figure 2), with few exceptions. Student’s t-tests and sign tests (see Additional file 10) revealed there were no statistically significant differences between the mean proportions for any residue at contact sites between mesophilic and thermophilic proteins. This is especially interesting because the overall proportion of mesophilic and thermophilic r-proteins differ in the residues R, I, Y, S and T—but these differences do not carry over into the identities of contact residues.

We further define a Contact Enrichment Factor (CEF) as the ratio between the percent of a given amino acid located at contact sites and the total amount of that residue present in the protein sequence. That is,

(1) CEF=RcCRtL

where L is the length of the protein (total number of residues), C is the estimated number of residues in contact with RNA in the fully assembled 30S subunit (see Methods), R_t_ is the total number of residues of a specific type (*e.g.* Alanine (A) or Serine (S)), and R_c_ is the number of contact residues of said type. CEF is closely related to the proportions of contact residues already reported (the numerator, R_c_/C, is the proportion of each residue as a contact, as described above), but CEF is not a redundant calculation, as it gives a broader measure of the role each amino acid plays in r-proteins. By comparing the fraction of a particular amino acid as a contact residue to its proportion in the total protein, CEF describes the distribution of each amino acid throughout the protein, revealing how often each residue is used as a contact site as a function of how often it occurs in the protein. Thus, a CEF value of 1 indicates that the residue under investigation appears at contact sites in the same percentage as it appears in the overall sequence, whereas CEF>1 implies that the residue has a high occurrence at the RNA contact interface for the proportion of that residue in the full protein.

We calculated CEF values of the r-proteins in all 560 species (Figure 5B). One-sample t-tests revealed that CEF values significantly deviated from one (two-tailed p < 0.01) for all the amino acids except for glycine, indicating that the distribution of amino acids in r-proteins is influenced by the interaction with rRNA. The results revealed that the mean contact enrichment factors were greater than 1 for positively charged residues and polar residues excluding Cysteine (C). CEF values were less than 1 for negatively charged and non-polar residues. These observations indicate that contact sites are generally enriched with positive and polar residues, which can form charge-charge or hydrogen bonding interactions, but are deficient of negative and non-polar residues, which might produce energetically unfavorable interactions with the rRNA. Contact enrichment factors for aromatic residues, which could participate in base-stacking with the rRNA nucleotides, were split: Phenylalanine (F) CEF was less than 1, whereas CEF for Tryptophan (W) and Tyrosine (Y) were greater than 1. It is worth noting that W and Y are both capable of hydrogen bonding, which could explain their preference at contact sites, but F is completely hydrophobic and is often found buried inside water-soluble proteins.

For the five amino acid chemical categories, the CEF for positively charged residues is the greatest, followed by polar residues, and those for negatively charged and nonpolar are lowest. This demonstrates that protein residues that contact rRNA tend to (1) carry a formal positive charge or contain a polar side chain and (2) avoid negatively charged or nonpolar residues. Therefore, not only do r-proteins contain a higher level of positively charged residues than non-ribosomal proteins, these residues are concentrated at rRNA contact sites. These general patterns reflect the role of positively charged regions of r-proteins in associating with the negatively charged rRNA during ribosomal assembly.

To test whether r-protein-rRNA interaction is different between mesophiles and thermophiles due to their differing overall amino acid compositions (as seen in Figure 2), we compared the CEF values between the two groups (Figure 5B). PIC indicated that most of those differences are not statistically significant (p > 0.01, Student’s t-test and sign test) except for Glutamic Acid (Glu, E; Figure 5B; see Additional file 11 for CEF statistical tests), which occurs at contact sites in one of the lowest proportions for both mesophiles and thermophiles (mean CEF = 0.43 and 0.37 for mesophiles and thermophiles, respectively), but is nonetheless statistically more common at mesophilic contact sites than thermophilic. Glu is not found in significantly different amounts in the overall composition of mesophilic and thermophilic r-proteins, and further investigation into Glu’s roles in the assembly process or thermostability in general might better explain this observation. The combination of significant thermostability-related differences in amino acid compositions (increased R, I, Y and decreased S, T for thermophiles) with no significant difference in the distribution of those amino acids at r-protein contact sites supports the understanding that the electrostatics component of ribosome assembly is not dependent on temperature, because the identity of thermophilic contact sites is statistically no different than that of mesophilic sites. This seems reasonable because other molecular interactions such as hydrogen bonding and hydrophobic interactions are sensitive to temperature, but the electrostatic interaction itself is independent of temperature, which likely explains why we observed similar amino acid residue distributions at the r-protein contact sites in mesophiles and thermophiles.

## Conclusion

Utilizing a comparative approach to analyze a large database of r-protein sequences has identified a number of important associations between the amino acid composition of r-proteins and their function in ribosomal assembly. We found that r-proteins have a significantly higher content of positively charged residues than do non-ribosomal proteins (10% for Arginine and 11% for Lysine in r-proteins, versus 4.7% and 5.9%, respectively, in non-ribosomal proteins), which agrees with previous analyses of r-protein charges. More specifically, these two residues are also highly represented at contact sites along the protein/RNA interface (contact enrichment factor (CEF) > 1) for all species in the study, alluding to the significance of electrostatic interaction in ribosome assembly. These results agree with and improve our previous r-protein study by statistically extending the same trends across a large sample of bacteria. Interestingly, we found that the percentage of Lysine residues generally increases with decreasing r-protein size, but the same correlation is not found with Arginine, despite its similar positively charged side chain. Taken together, these results corroborate the heavy emphasis on electrostatic interactions in the assembly mechanism of the ribosome. However, association between r-protein binding order (primary, secondary, and tertiary) was not detected for the proportion of positively charged residues (or Lys or Arg alone) or for net protein charge. This leads to the conclusion that the order in which r-proteins bind to their binding sites during assembly is probably not determined by the electrostatics interactions between r-proteins and rRNA. Although the assembly between r-proteins with rRNA involves an overwhelmingly significant portion of electrostatic interaction, this interaction alone does not govern the assembly order.

The thermostability aspect of the study, performed by comparing amino acid compositions and distributions between species with high and low preferred growth temperature, revealed two noteworthy characteristics of 30S ribosomal proteins. First, we found that thermophiles show increased R, I, and Y content, whereas mesophiles have increased proportions of S and T, trends that are generally consistent with previously reported distinctions between thermophilic and mesophilic amino acid compositions [29]. Second, while these differences in overall make-up are significant, they do not extend to the predicted contact sites in thermophilic and mesophilic r-proteins. That is, the proportions of residues at contact sites are generally not significantly different between the two groups. Whereas the percent compositions of amino acids relating to qualities such as thermostability and protein folding are expected to vary with environmental temperature, our results indicate that the distributions of residues in contact with rRNA are comparable for all bacterial species. If the regions of r-proteins that contact rRNA in the fully assembled ribosome are considered “active sites” for the assembly process, it follows that they should be as highly conserved as the ribosome and its function themselves. In accordance, from the results of the current study, we conclude that the electrostatics component of ribosome assembly, while it is not the only interaction involved during assembly, is an important attraction between r-proteins and rRNA, but this component of interaction is insensitive to the temperature. The latter conclusion is reasonable because the electrostatics interaction itself does not depend on temperature.

Therefore, we conclude from our statistical analysis: binding order does not appear to depend on the amount of electrostatic attraction experienced by primary binders versus secondary or tertiary binders, and the electrostatics interactions of ribosome assembly do not seem to control the discrepancy between mesophilic temperature-sensitive and thermophilic high-temperature-stable constructs. The particular molecular factors that govern the timing and order of r-proteins binding with rRNA and that contribute to the temperature sensitivity of ribosomes assembled in species that live at different temperatures remain to be determined.

## Methods

### Study samples

The study required three pieces of information for each of 560 bacterial species: growth temperature preference (mesophilic or thermophilic), amino acid composition data based on amino acid sequences of 30S ribosomal proteins, and 16S ribosomal DNA sequences for the phylogenetic tree construction required for PIC. We only included species with all three pieces of information publicly available. Estimates of the growth temperature preference of studied species were searched based on the species name and obtained from various sources in the public domain. Initially, species were categorized into four growth temperature preference types; cryophiles (e.g., high latitude, altitude habitats, ocean floor, < 10°C), lower mesophiles (ambient conditions, 10-35°C), upper mesophiles (e.g., mammalian body, 35-50°C), and thermophiles (e.g., deep see thermal vents, hot springs, >50°C). Examination of the distribution of amino acid composition based on these four categories indicated that the distributions of the first three categories were often similar to each other but markedly different from that of thermophiles, particularly for positive and polar residues. Therefore, we combined species in the first three categories and conducted subsequent analyses using only two categories; mesophiles (<50°C) and thermophiles (>50°C).

### 30S ribosomal protein sequences

Amino acid sequences for the S2-S21 30S ribosomal protein were queried and downloaded from Genbank (http://www.ncbi.nlm.nih.gov/) using the search term “30S ribosomal protein”. Protein S1 was excluded from the analysis, as in many other 30S ribosomal protein studies, because it binds relatively weakly to the 30S complex and exchanges very rapidly during protein assembly [43]. The queried sequences were aligned using the T-coffee multiple alignment program [44] (http://www.tcoffee.org/Projects_home_page/t_coffee_home_page.html) using default alignment settings. We filtered out potentially spurious sequences that 1) were unusually short or long and 2) had unusually low T-coffee alignment scores, which might indicate poor sequence quality or incorrect genes. When multiple sequences from the same species were available, we chose the one with the highest alignment score. Gaps and missing sequences were ignored in the subsequent analyses.

### Non-ribosomal protein sequences

To compare the amino acid proportions of ribosomal and non-ribosomal proteins, we analyzed protein sequences of 15 house-keeping protein families that are functionally well-defined and distinct from each other: adenylate kinase, carbamoyltransferase, carboxypeptidase, citrate synthase, ferredoxin, glutamate dehydrogenase, glycosyltransferase, inorganic pyrophosphatase, methionine aminopeptidase, phosphofructokinase, phosphoglycerate kinase, reductase, rubredoxin, triose phosphate isomerase, xylanase. Their sequences were queried and downloaded from Genbank by using each protein name along with the name of each of the 560 species used for the ribosomal proteins analyses as search terms. (See Additional file 12 for the number of species for and Additional file 13 for a description of each protein family used in this study.) The first sequence returned in each search was used for the analyses. When no sequence was available for a given species, the species was omitted from the analysis for that protein. Student’s paired sample t-test was performed to test the equality of the amino acid distributions between ribosomal and each non-ribosomal protein.

### Determination of ribosomal protein-RNA contact sites and protein net charge

The r-protein/rRNA contact sites were obtained from the *E. coli*[41] [PDB: 2AVY] and *T. thermophilus*[42] [PDB: 1J5E] 30S x-ray crystal structures, accessed from the Protein Data Bank [45]. Using a code written in our own group as described in our previous r-protein study [12], any atom on a protein residue within 3.5Å of any atom on a 16S rRNA nucleotide is considered a contact point. A contact residue is a protein residue that makes at least one contact point with any RNA nucleotide. The identity and position of these contact residues found in the assembled 30S subunit were recorded and used for further analysis. Because the rRNA contact sites of *E. coli* and *T. thermophilus* are not always conserved, we designated rRNA contact sites of all the studied species based on the shared contact sites between these two reference species. These contact sites, therefore, should be considered conservative. Protein net charge was calculated according to the formula [(K + R) – (D + E)], where (K+R) represents the number of Lysine and Arginine residues (positively charged) and (D+E) represents the number of Aspartic Acid and Glutamic acid residues (negatively charged). All other residues are considered neutrally charged at physiological pH.

### 16S rDNA sequences and phylogenetic tree construction

To construct a phylogenetic tree required for PIC, we queried bacterial 16S rDNA sequences based on the species name from Greengenes database (greengenes.lbl.gov), which curates and aligns publicly available prokaryotic 16S ribosomal RNA gene sequences. Based on the sequence alignments from Greengenes, we constructed a majority-rule consensus phylogenetic tree of the studied species using MrBayes [46] (http://mrbayes.sourceforge.net), which uses Markov Chain Monte Carlo (MCMC) methods to estimate Bayesian inference of evolutionary relationships. We used Modeltest [47] to search for a nucleotide substitution model that fit our dataset and selected GTR+G (General Time Reversible with gamma-shaped rate variation among sites) with a flat Dirichlet prior probability density, evaluated based on Akaike Information Criterion (AIC).

### Phylogenetic Independent Contrast (PIC)

To assess the association between growth temperature preference of bacterial species and their amino acid composition using PIC, we used the AOT module of Phylocom [48] (http://www.phylodiversity.net/phylocom/), incorporating the branch lengths in the Bayesian tree. Each protein contained an overlapping but different set of species sequences from other proteins. Therefore, when proteins are analyzed separately for PIC, the original phylogenetic tree was pruned using the ‘sampleprune’ module of Phylocom to filter out missing species. When a binary trait is involved in a PIC analysis (as for growth temperature preference in this study, i.e., mesophile or thermophile), AOT identifies independently contrasting tree nodes based on a combination of both the sister-taxa (ST) set and the paraphyletic (PT) set, and calculates trait correlations using these independent contrasts. Significance of independent contrasts was tested using two separate tests; t-test and sign test. In t-test, the mean and standard deviation of the contrasts were used to conduct a one-sample t-test with degree of freedom of N (number of contrasts) - 1 against the null hypothesis of mean = 0. In sign test, binomial probabilities were calculated for the number of contrasts toward one direction against the total number of contrasts.

### Statistical tests

Student’s paired-sample and one-sample t-tests, Pearson’s product–moment and Spearman’s rank correlations, Pearson’s χ^2^ tests, and descriptive statistics including box plots were calculated using PASW Statistics18 (IBM, New York, NY) and R (http://www.r-project.org/). Two-tailed Fisher’s exact tests were conducted using the Fisher’s exact test Excel Addin (http://www.obertfamily.com/software/fisherexact.html). Effect size of Fisher’s exact tests was estimated using the ϕ^2^ coefficient (ϕ^2^ = √ (χ2/N), where N is the number of samples).

## Availability of supporting data

The data sets supporting the results of this article are included within the article and its additional files.

## Competing interests

The authors declare that they have no competing interests.

## Authors’ contributions

BBL, YW and TN designed the study, BBL and TN performed analysis, and BBL, YW and TN wrote the manuscript. All authors read and approved the final manuscript.

## Supplementary Material

Additional file 1 **Bacterial species under investigation.** Unrooted 50% majority rule consensus Bayesian phylogenetic tree of the 560 bacterial species used in the study. The length of the bottom bar indicates 0.1 substitution per nucleotide. The letter in front of each species name indicates growth temperature preference (M: mesophiles, T: thermophiles). Thermophilic species are boxed in red. Click here for file

Additional file 2 Student’s T-test t- and p-values for r-protein versus non-ribosomal protein amino acid compositions (α=0.01). Click here for file

Additional file 3 Statistical test results for mesophile/thermophile amino acid composition differences (α=0.01). Click here for file

Additional file 4 **Percentage of positively charged residues and net charges for mesophilic and thermophilic r-proteins, arranged according to binding order.** This figure presents the same data as Figure 3 of the manuscript, but more clearly shows that no relationship exists between binding order and percent of positive residues or net protein charge. All thermophilic r-proteins except S11 contain a higher percentage of positively charged residues than their mesophilic homologs (a), and, for some proteins, including all six primary proteins, this difference is statistically significant. R-proteins generally have a net positive charge (b), and thermophiles typically have a higher average charge than mesophiles. For three proteins, this difference is significant. In the box-and-whisker representation, the lower and upper circles represent the 5th and 95th percentiles, respectively, and the lower and upper whiskers the 10th and 90th. The colored regions mark the middle 50% of the samples (25th to 75th percentile), with a solid line representing the median and a dotted line the mean. Asterisks mark the amino acids that show a statistically significant difference between mesophilic and thermophilic species.kb) Click here for file

Additional file 5 **Ribosomal protein composition summaries.** Amino acid compositions for mesophilic (blue) and thermophilic (red) r-proteins according to chemical property: (a) polar, (b) negative, (c) nonpolar, and (d) aromatic residues. In the box-and-whisker representation, the lower and upper circles represent the 5th and 95th percentiles, respectively, and the lower and upper whiskers the 10th and 90th. The colored regions mark the middle 50% of the samples (25th to 75th percentile), with a solid line representing the median and a dotted line the mean. Asterisks mark the amino acids that show a statistically significant difference between mesophilic and thermophilic species. Click here for file

Additional file 6 Statistical test results for proportions of mesophile/thermophile amino acid residues by property (α=0.01). Click here for file

Additional file 7 Statistical test results for mesophile/thermophile r-protein net charges (α=0.01). Click here for file

Additional file 8 **Spearman’s Rank Correlation (ρ, *rho*) and p-values between amino acid and r-protein length (α=0.01).** Click here for file

Additional file 9 **Proportion of Lys and Arg as a function of average protein length.** Another representation of the relationship depicted in Figure 4, this figure plots Lys proportion and Arg proportion against the average length of r-protein, showing the significant, strong inverse correlation with Lys and the weaker, insignificant correlation with Arg. Click here for file

Additional file 10 Statistical test results for mesophile/thermophile CEF differences (α=0.01). Click here for file

Additional file 11 **Statistical test results for mesophile/thermophile R**_**c**_**/C values (α=0.01).** Click here for file

Additional file 12 Protein families under investigation. Click here for file

Additional file 13 Biochemical description of non-ribosomal protein families. Click here for file
