# Supplementary figures and images for: A comparative study of ribosomal proteins: linkage between amino acid distribution and ribosomal assembly
Source: BMC Biophys. 2013 Oct 23;6:13. doi: 10.1186/2046-1682-6-13 (PMC4016315; doi:10.1186/2046-1682-6-13)

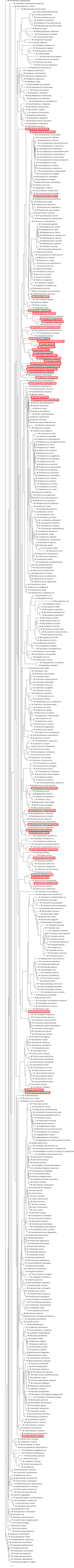

Supplement: Additional file 1 — Bacterial species under investigation. Unrooted 50% majority rule consensus Bayesian phylogenetic tree of the 560 bacterial species used in the study. The length of the bottom bar indicates 0.1 substitution per nucleotide. The letter in front of each species name indicates growth temperature preference (M: mesophiles, T: thermophiles). Thermophilic species are boxed in red. [file 2046-1682-6-13-S1.pdf]

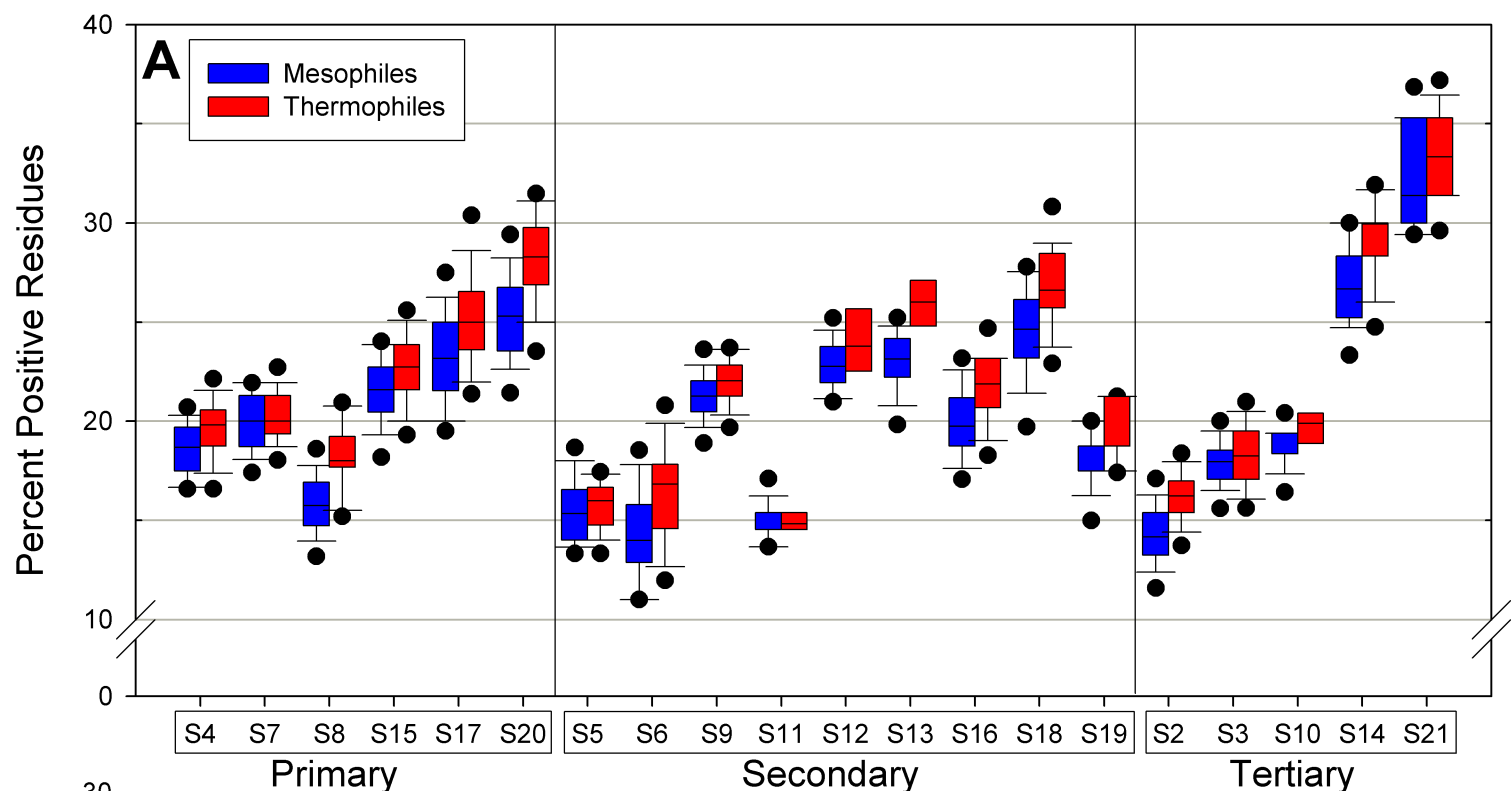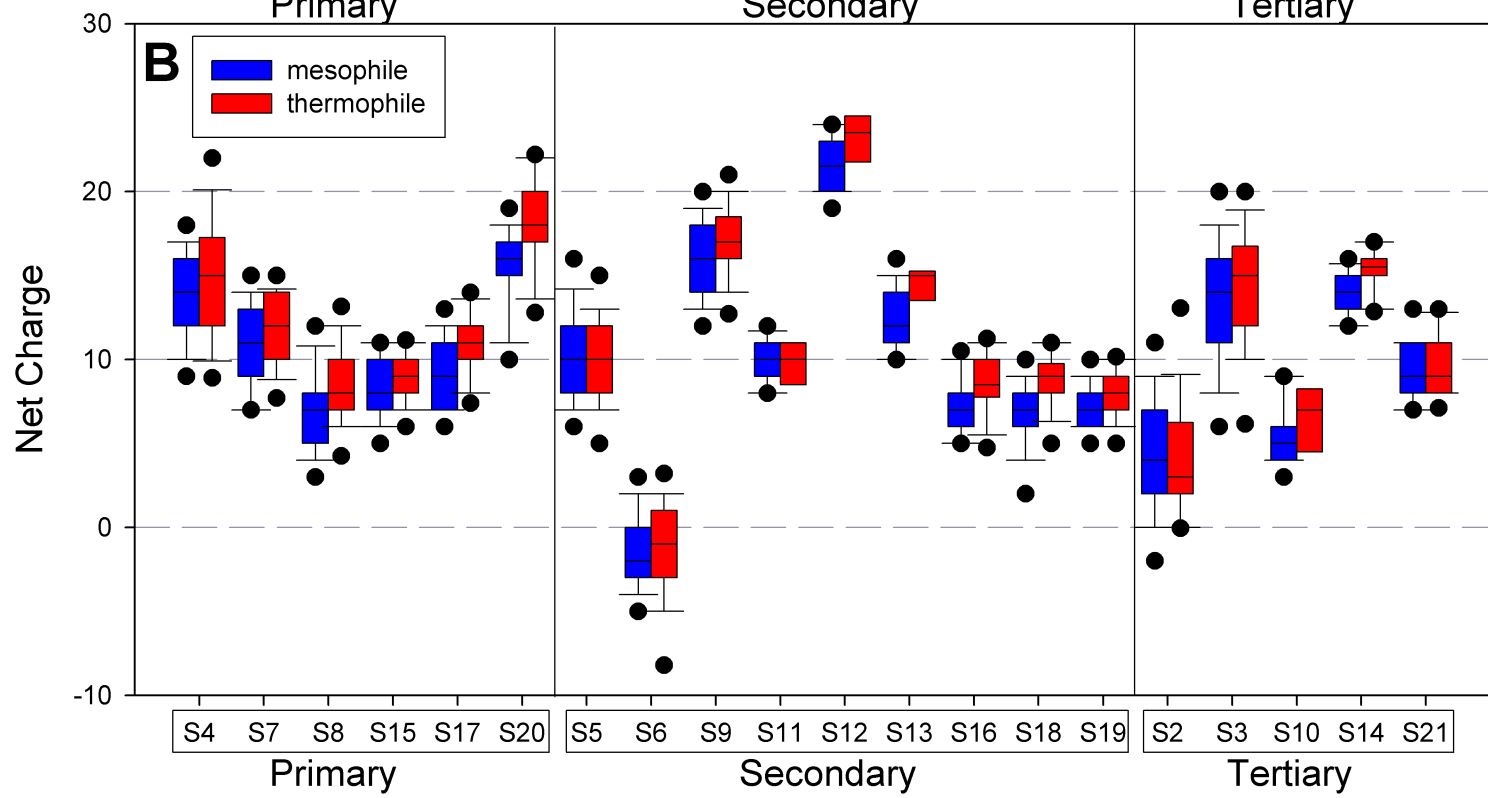

Supplement: Additional file 4 — Percentage of positively charged residues and net charges for mesophilic and thermophilic r-proteins, arranged according to binding order. This figure presents the same data as Figure 3 of the manuscript, but more clearly shows that no relationship exists between binding order and percent of positive residues or net protein charge. All thermophilic r-proteins except S11 contain a higher percentage of positively charged residues than their mesophilic homologs (a), and, for some proteins, including all six primary proteins, this difference is statistically significant. R-proteins generally have a net positive charge (b), and thermophiles typically have a higher average charge than mesophiles. For three proteins, this difference is significant. In the box-and-whisker representation, the lower and upper circles represent the 5th and 95th percentiles, respectively, and the lower and upper whiskers the 10th and 90th. The colored regions mark the middle 50% of the samples (25th to 75th percentile), with a solid line representing the median and a dotted line the mean. Asterisks mark the amino acids that show a statistically significant difference between mesophilic and thermophilic species.kb) [file 2046-1682-6-13-S4.pdf]

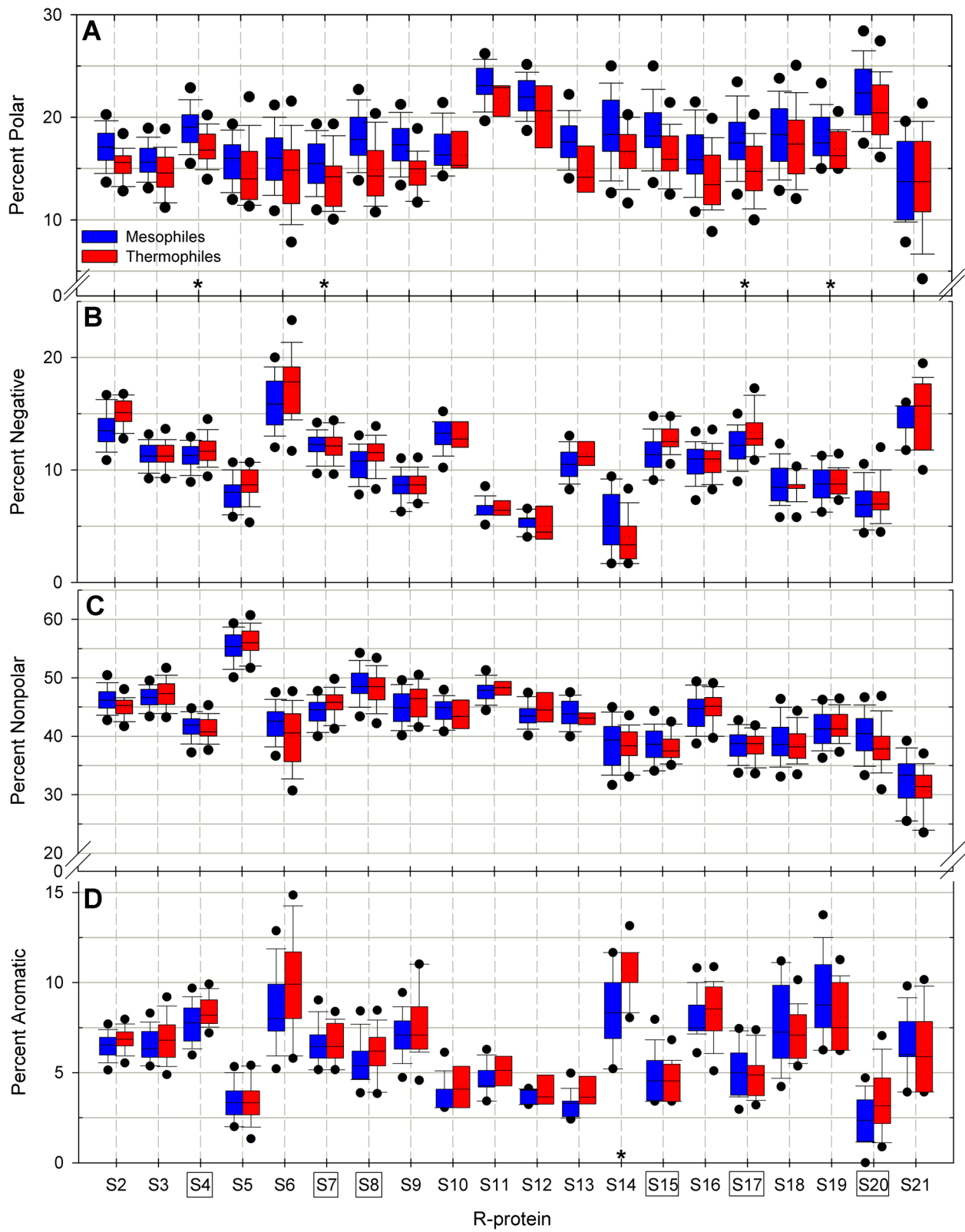

Supplement: Additional file 5 — Ribosomal protein composition summaries. Amino acid compositions for mesophilic (blue) and thermophilic (red) r-proteins according to chemical property: (a) polar, (b) negative, (c) nonpolar, and (d) aromatic residues. In the box-and-whisker representation, the lower and upper circles represent the 5th and 95th percentiles, respectively, and the lower and upper whiskers the 10th and 90th. The colored regions mark the middle 50% of the samples (25th to 75th percentile), with a solid line representing the median and a dotted line the mean. Asterisks mark the amino acids that show a statistically significant difference between mesophilic and thermophilic species. [file 2046-1682-6-13-S5.pdf]

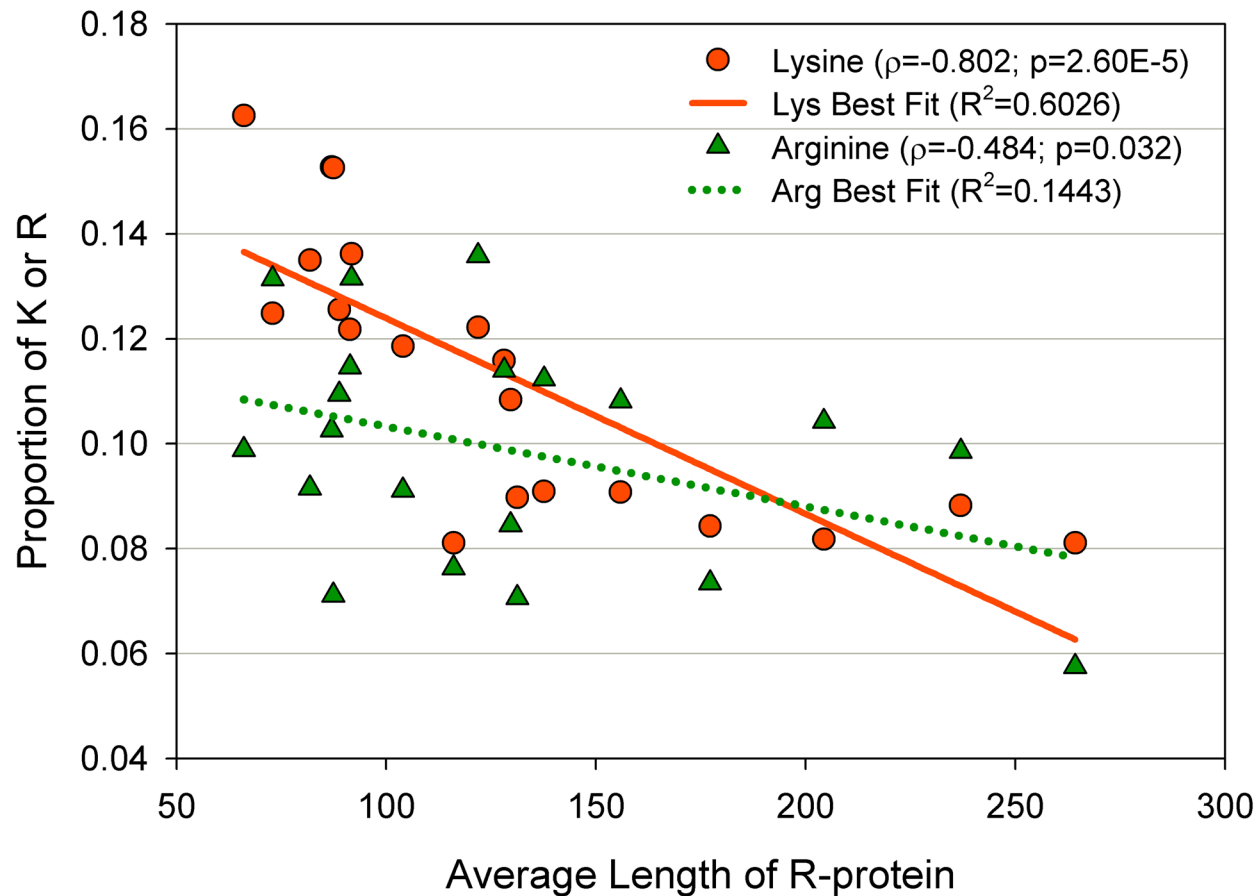

Supplement: Additional file 9 — Proportion of Lys and Arg as a function of average protein length. Another representation of the relationship depicted in Figure 4, this figure plots Lys proportion and Arg proportion against the average length of r-protein, showing the significant, strong inverse correlation with Lys and the weaker, insignificant correlation with Arg. [file 2046-1682-6-13-S9.pdf]
