# Supplementary material for: A comparative study of ribosomal proteins: linkage between amino acid distribution and ribosomal assembly
Source: BMC Biophys. 2013 Oct 23;6:13. doi: 10.1186/2046-1682-6-13 (PMC4016315; doi:10.1186/2046-1682-6-13)
Supplement: Additional file 13 — Biochemical description of non-ribosomal protein families. [file 2046-1682-6-13-S13.pdf]

Supplementary Material

Protein Families

Adenylate kinase plays an important role in maintaining the level of ATP in bacteria by catalyzing the reversible reaction $2ADP \leftrightarrow ATP + AMP$ [1]. Inorganic pyrophosphatase enzymes regulate the level of free phosphates available to the cell via the energetically favorable hydrolysis of the inorganic phosphate anhydride (PP_i) bond[1]. Glycosyl transferase is a highly conserved enzyme unique to bacteria which helps synthesize the peptidoglycan cell wall in Gram positive organisms[2]. Carboxypeptidase and Methionine aminopeptidase are families of enzymes which hydrolyze peptide bonds: at the carboxyl terminus of a polypeptide and by cleaving the amino terminal methionine residue, respectively[1]. Glutamate dehydrogenase participates in a number of highly regulated metabolic pathways, including both carbon and nitrogen fixation, and catalyzes the interconversion between glutamate and α-ketoglutarate, using either NAD^+ or $NADP^+$ as an acceptor[1]. Xylanase refers to a family of enzymes which work to break glycosidic bonds of a plant-based polysaccharide called xylan, which often contains high levels of the monosaccharide xylose[3]. Phosphofructokinase (PFK), phosphoglycerate kinase, and triose phosphate isomerase are three classes of enzymes primarily known for their roles in glycolysis. PFK and phosphoglycerate kinase catalyze the transfer of a phosphate group between ATP and their metabolic substrates: fructose 6-phosphate and bisphosphoglycerate, respectively. Triose phosphate isomerase converts dihydroxyacetone phosphate to glyceraldehydes 3-phosphate in the last step in the preparatory phase of glycolysis.[1] Citrate synthase catalyzes the exergonic first step of the Citric Acid Cycle by hydrolyzing acetyl Coenzyme A (CoA) to form citrate and free CoA[1]. Ferredoxin[4] and rubredoxin[5] are non-heme iron-based enzymes which function in electron transport pathways, including hydrogen production, nitrogen fixation, sulfur reduction, and photosynthesis. Reductase is a general term for an enzyme which catalyzes a reduction reaction, and carbamoyltransferase catalyzes the transfer of a carbamoyl group, often between carbamoyl phosphate and small organic molecules such as aspartate and ornithine.

1. Lehninger AL, Nelson DL, Cox MM: *Lehninger principles of biochemistry.* 5th edn. New York: W.H. Freeman; 2008.
2. Lovering AL, de Castro LH, Lim D, Strynadka NC: **Structural insight into the transglycosylation step of bacterial cell-wall biosynthesis.** *Science* 2007, **315:**1402-1405.
3. Beg QK, Kapoor M, Mahajan L, Hoondal GS: **Microbial xylanases and their industrial applications: a review.** *Appl Microbiol Biotechnol* 2001, **56:**326-338.
4. Valentine RC: **Bacterial Ferredoxin.** *Bacteriol Rev* 1964, **28:**497-517.
5. Adman ET, Sieker LC, Jensen LH: **Structure of rubredoxin from Desulfovibrio vulgaris at 1.5 A resolution.** *J Mol Biol* 1991, **217:**337-352.
